# Long-Lasting SARS-CoV-2 Infection With Post-COVID-19 Chronic Interstitial Pneumonia in a Patient With Chronic Lymphocytic Leukemia Treated Successfully With Intravenous Immunoglobulin

**DOI:** 10.7759/cureus.51890

**Published:** 2024-01-08

**Authors:** Ahmad Haddad, Qusai Al-maharmeh, Mohammad N Kloub, Elrazi A Ali, Hamid Shaaban

**Affiliations:** 1 Internal Medicine, Saint Michael's Medical Center, Newark, USA; 2 Internal Medicine, One Brooklyn Health/Interfaith Medical Center, Brooklyn, USA; 3 Hematology/Oncology, Saint Michael's Medical Center, Newark, USA

**Keywords:** intravenous immunoglobulin, non-specific interstitial pneumonia, chronic lymphocytic leukemia (cll), sars-cov-2 and covid-19, covid-19 in cancer patients

## Abstract

Post-coronavirus disease 2019 (post-COVID-19) condition is a post-acute syndrome characterized by non-specific symptoms that remain for at least two months and typically appear three months after the start of the acute phase. Individuals with chronic lymphocytic leukemia (CLL) are considered to be at high risk of contracting COVID-19. It is also becoming increasingly evident that the severe acute respiratory syndrome coronavirus 2 (SARS-CoV-2) vaccine response is frequently lacking or insufficient. We present a 77-year-old male patient with CLL who had multiple hospitalizations for the management of pneumonia related to persistent COVID-19 infection due to hypogammaglobulinemia. He was subsequently treated with intravenous immunoglobulin (IVIG). This case emphasizes the importance of the early detection of hypogammaglobulinemia in patients with CLL and long COVID because of the potential therapeutic benefit of IVIG therapy. We also provide a literature review on COVID-19 infection in CLL patients, focusing mainly on the subset population of patients with hypergammaglobulinemia.

## Introduction

The coronavirus disease 2019 (COVID-19) started in 2019 and has been a challenging infection throughout all aspects of medicine. For some patients, it has manifested as severe and life-threatening pneumonia, particularly in the elderly and leukemia patients [1,2]. As a result of the treatment or the leukemia itself, cancer patients are more vulnerable to the complications of COVID-19 infection. This is caused by the morbidity associated with the cancer or the cytotoxic medications that are being used for treating leukemia [2]. The relationship between hypoglobulinemia and viral infection is not well studied, but some studies show that patients with hypoglobulinemia are more prone to recurrent and persistent viral infections [3]. In this case, we are presenting a chronic lymphocytic leukemia (CLL) patient who has been having worsening symptoms and shortness of breath and has been continuously positive for COVID-19 polymerase chain reaction (PCR), and his CT scan shows worsening of infiltrations as he was found to have low immunoglobulin levels. The patient subsequently clinically improved after being given intravenous immunoglobulin (IVIG), and his symptoms resolved afterward.

## Case presentation

A 77-year-old non-smoker male, fully vaccinated against COVID-19, with a past medical history of stage 3 CLL diagnosed 13 years ago according to the modified Rai clinical staging system for CLL and who had six cycles of venetoclax 300 mg daily maintenance, presented with complaints of progressive shortness of breath for four months' duration after he was diagnosed with COVID-19 infection, despite being treated with an antiviral and steroids at the time of diagnosis. 

The patient's shortness of breath worsens with time, with COVID-19 PCR tests being persistently positive on multiple occasions. The first test was positive four months before this presentation, the second one was three months before this presentation, and the third one was two months before this presentation when he started requiring 2 L of oxygen to ambulate. Shortness of breath was associated with a productive cough, and the patient could not walk a few steps without rest. Blood cultures on the second and third presentations (three and two months before this presentation) were negative. On examination, the patient was vitally stable but required oxygen to maintain saturation; a chest examination showed fine bilateral crackles without lower limb edema or jugular venous distension.

Initial labs showed normal complete blood count, complete metabolic panel, and brain natriuretic peptide. Procalcitonin was negative, but C-reactive protein (CRP) was 11 mg/L (normal range: 0-0.8 mg/L). COVID-19 PCR test was positive. The respiratory viral panel came back negative apart from COVID-19, with negative *Pneumocystis jirovecii* PCR test.

**Figure 1:**
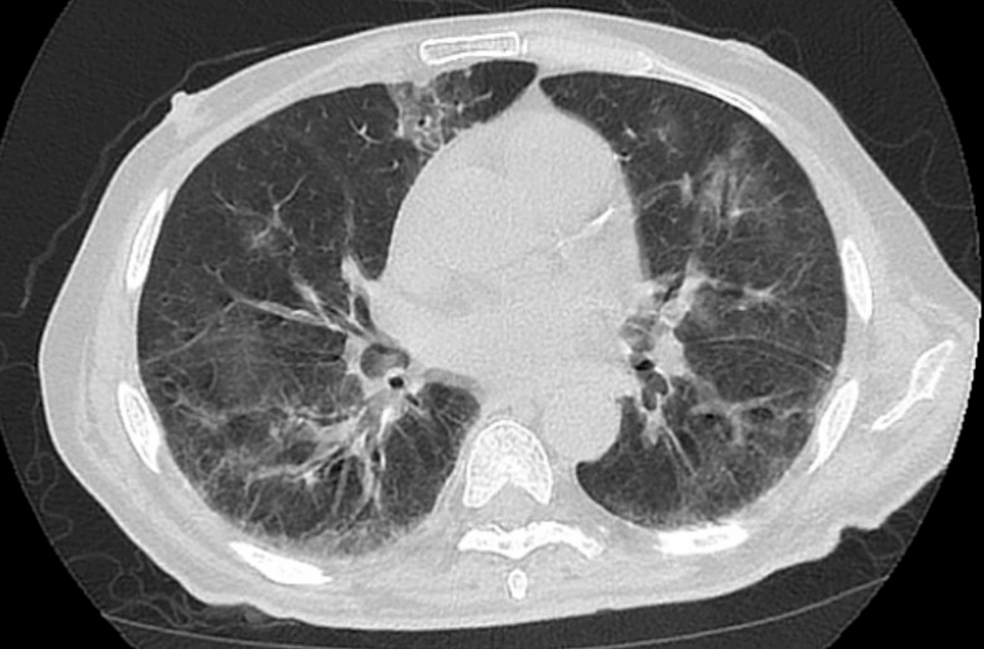
Scattered peripheral and basilar ground-glass infiltrates were worse when compared to previous images (at the time of presentation).

**Figure 2:**
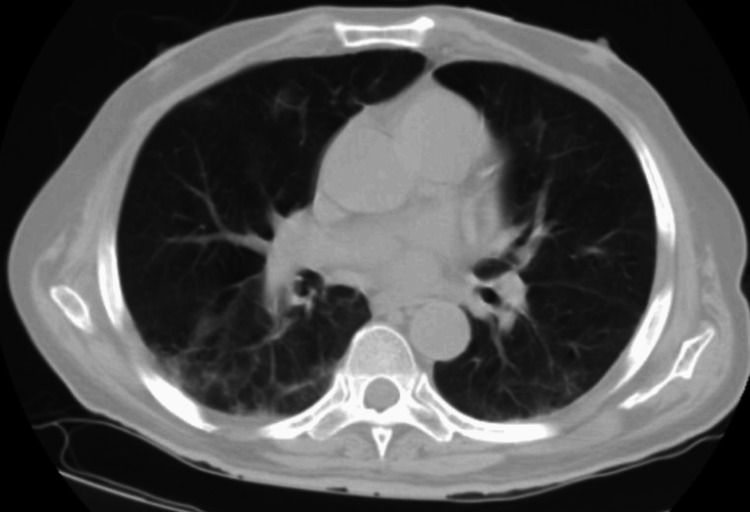
Scattered bilateral ground-glass opacities with bibasilar linear segmental atelectasis (three months before presentation).

**Figure 3:**
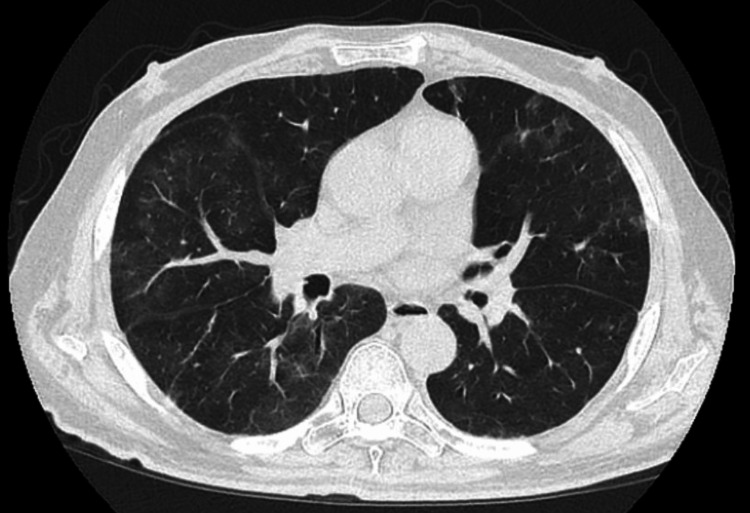
Bilateral ground-glass infiltrates (four months before presentation).

CT scan showed scattered peripheral ground-glass infiltrates (Figure 1), which is worse when compared with previous CT scans done three and four months before (Figure 2 and Figure 3, respectively). The patient was again started on IV steroids and molnupiravir with no improvement in his symptoms over the next few days, so immunoglobulin levels were ordered and showed IgA was 13 mg/dl (normal value: 61-437 mg/dl), IgG 109 mg/dl (normal value: 603-1613 mg/dl), and IgM 15 mg/dl (normal value: 15-143 mg/dl).

The patient was started on IVIG weight-based dose and showed significant improvement after the first dose. Two weeks later, his shortness of breath totally improved, and he does not require oxygen anymore. His COVID-19 PCR test returned negative, despite not holding rituximab and venetoclax during his treatment duration.

## Discussion

By the end of 2019, it was determined that a new coronavirus began a series of worldwide epidemics that started in China. It then evolved to be a pandemic after it caused a cluster of pneumonia cases [4]. As of December 19, 2023, the number of cases in the United States was 103,436,829, including 1,144,877 deaths. The number of confirmed cases around the world was 772,838,745, which includes 6,988,679 deaths as reported by the WHO [5]. Cancer patients are typically more susceptible to complications and associated with worse clinical outcomes of COVID-19 than patients without cancer [6].

Coronavirus is an enveloped positive-stranded RNA virus. Direct person-to-person respiratory transmission is the primary way to get infected with COVID-19 [7]. By directly detecting SARS-CoV-2 RNA using the nucleic acid amplification test (NAAT) or by detecting viral protein using an antigen test, COVID-19 can be diagnosed. Generally, a positive NAAT or antigen test indicates infection and does not require repeat testing. The median duration for SARS-CoV-2 viral shedding from the onset of symptoms has been documented to be around 12-20 days [8]. Cancer patients had a 60% increased likelihood of a positive COVID-19 test result than people without the disease [9]. It has also been demonstrated that individuals diagnosed with cancer have higher rates of observed mortality, ICU hospitalizations, invasive ventilation requirements, and the development of at least one severe or critical symptom. These statistics are accurate for hematological malignancies such as lymphomas, leukemia, and myelomas [10]. CLL patients, in particular, have a high risk for a poor outcome from COVID-19 infection, and this can be attributed to co-existing low immunoglobulin levels caused by CLL itself or the treatment regimens which include rituximab [11].

CLL is a chronic lymphoproliferative disorder that occurs when dysfunctional or incompetent monoclonal B lymphocytes accumulate. It is the most common leukemia in adults in the United States, accounting for 25-35% of all leukemias in the United States [12]. Generally, CLL is an asymptomatic disease; most of the cases will be found incidentally on blood work when it shows lymphocytosis, and only 5-10% of people present with symptoms such as fever, night sweats, weight loss, and fatigue [3]. Some abnormal findings can be found in CLL patients; one of them is hypogammaglobulinemia. Twenty-five percent of people at the time of being diagnosed with CLL have hypogammaglobulinemia, and up to two-thirds will have it later in the disease's course. Usually, IgM, IgG, and IgA are decreased; in some patients, we can see two or one of them is decreased [13].

The connection between COVID-19 infection and hypogammaglobulinemia status is not well understood at this time. CLL patients are more vulnerable to viral respiratory infections; this can be due to low immunoglobulin levels, mainly the IgA, which is the main immunoglobulin in the respiratory tract which is important to prevent the binding of microbial agents; this low immunoglobulin status makes mucosal immune deficit [14].

Biologics are the mainstay treatment in the management of CLL. Despite the evolving treatment paradigm, rituximab (in combination with chemotherapy) will likely retain a significant role in CLL. However, this could also hasten B-cell exhaustion, leading to a clinically noteworthy decline in immunoglobulin levels and insufficient immune response to immunizations [15]. Up to 6.6% of patients may develop chronic hypogammaglobulinemia as a result of anti-CD20 medication, which is associated with a greater risk of infection [16].

COVID-19 infection is usually treated depending on the severity of the disease and symptoms, ranging from conservative and supportive measurements to antiviral agents and steroids. There are specific indications for treating patients with antiviral agents [17]. However, as far as we know, we did not find any case in the literature mentioning patients continuously testing positive for COVID-19 after being treated with the standard intervention but still have worsening symptoms.

Immunoglobulin therapy is indicated when we have impaired B cells, as in the case of post transplantation, multiple myeloma, and CLL, especially when using rituximab or other T cell-directed therapy against CD19 [18]; for patients with severe or recurrent pneumonia or those with concurrent acute illnesses like sepsis, IVIG treatment is typically recommended initially. In the third and fourth weeks following IVIG therapy, 10-15% of patients with an elevated risk of infection may benefit from immunoglobulin administration [19]. In our case, our patient had a long course of COVID-19 infection with underlying CLL and hypogammaglobulinemia status and was clinically not responding to the standard therapy of COVID-19. This was likely attributed to the severe hypogammaglobulinemia with the inability to produce neutralizing antibodies.

Our patient had long complained of respiratory symptoms with COVID-19 pneumonia because of the lack of development of an effective anti-SARS-CoV-2 plasma humoral response, and he showed persisting positive nasopharyngeal reverse transcription (RT)-PCR for SARS-CoV-2. Proal and VanElzakker suggested that SARS-CoV-2 may result in multiple chronic symptoms because it persists in different tissue reservoirs after acute infection, as confirmed by the identification of SARS-CoV-2 inert viral RNA and proteins. It is also increasingly clear that SARS-CoV-2 preferentially infects nasal epithelial cells, as already demonstrated by the high levels of angiotensin-converting enzyme 2 (ACE2) expression in this cell type [20].

## Conclusions

Persistent COVID-19 infection and its treatment in CLL patients are not well studied. CLL patients may present low immunoglobulin levels secondary to CLL or rituximab treatment. This will undoubtedly increase their risk of developing viremia and persistent viral infections that may not be responsive to the standard treatment. Vaccines also have ineffective humoral responses and, hence, lower efficacy in these patients. In conclusion, the use of IVIG, long considered only an ancillary therapy in individuals with CLL on long-term/continuous therapies, must be carefully taken into consideration by physicians who manage these patients with COVID-19 infections, given the longer life expectancy.
